# Line Scan Raman Microspectroscopy for Label-Free Diagnosis of Human Pituitary Biopsies

**DOI:** 10.3390/molecules24193577

**Published:** 2019-10-04

**Authors:** Daniela Bovenkamp, Alexander Micko, Jeremias Püls, Fabian Placzek, Romana Höftberger, Greisa Vila, Rainer Leitgeb, Wolfgang Drexler, Marco Andreana, Stefan Wolfsberger, Angelika Unterhuber

**Affiliations:** 1 Center for Medical Physics and Biomedical Engineering, Medical University of Vienna, Waehringer Guertel 18-20, 1090 Vienna, Austria; daniela.bovenkamp@meduniwien.ac.at (D.B.); jeremias.puels@meduniwien.ac.at (J.P.); fabian.placzek@meduniwien.ac.at (F.P.); rainer.leitgeb@meduniwien.ac.at (R.L.); wolfgang.drexler@meduniwien.ac.at (W.D.); angelika.unterhuber@meduniwien.ac.at (A.U.); 2 Department of Neurosurgery, Medical University of Vienna, Waehringer Guertel 18-20, 1090 Vienna, Austria; alexander.micko@meduniwien.ac.at (A.M.); stefan.wolfsberger@meduniwien.ac.at (S.W.); 3 Institute of Neurology, Medical University of Vienna, Waehringer Guertel 18-20, 1090 Vienna, Austria; romana.hoeftberger@meduniwien.ac.at; 4 Department of Internal Medicine III, Division of Endocrinology and Metabolism, Medical University of Vienna, Waehringer Guertel 18-20, 1090 Vienna, Austria; greisa.vila@meduniwien.ac.at

**Keywords:** raman spectroscopy, line scan raman microspectroscopy, pituitary gland, pituitary adenoma, principal component analysis, k-nearest neighbor classifier, texture analysis, grey level cooccurrence matrix, correlation coefficients

## Abstract

Pituitary adenomas are neoplasia of the anterior pituitary gland and can be subdivided into hormone-producing tumors (lactotroph, corticotroph, gonadotroph, somatotroph, thyreotroph or plurihormonal) and hormone-inactive tumors (silent or null cell adenomas) based on their hormonal status. We therefore developed a line scan Raman microspectroscopy (LSRM) system to detect, discriminate and hyperspectrally visualize pituitary gland from pituitary adenomas based on molecular differences. By applying principal component analysis followed by a k-nearest neighbor algorithm, specific hormone states were identified and a clear discrimination between pituitary gland and various adenoma subtypes was achieved. The classifier yielded an accuracy of 95% for gland tissue and 84–99% for adenoma subtypes. With an overall accuracy of 92%, our LSRM system has proven its potential to differentiate pituitary gland from pituitary adenomas. LSRM images based on the presence of specific Raman bands were created, and such images provided additional insight into the spatial distribution of particular molecular compounds. Pathological states could be molecularly differentiated and characterized with texture analysis evaluating Grey Level Cooccurrence Matrices for each LSRM image, as well as correlation coefficients between LSRM images.

## 1. Introduction

The main function of the pituitary gland, which is situated under the base of the brain, is the production of hormones. Owing to a feedback mechanism, the pituitary gland is responsible for regulating the endocrine glands within the body and thereby regulation of organ function. Hormones which are produced by this gland include growth hormone (hGH), adrenocorticotropic hormone (ACTH), thyroid-stimulating hormone (TSH), gonadotropins (LH/FSH) and prolactin (PRL). Neoplasia of the anterior pituitary gland, or pituitary adenomas, represent approximately 15% of intracranial tumors [1,2]. Pituitary adenomas are specified as hormone-producing tumors and categorized according to either the hormone which is overexpressed (lactotroph, corticotroph, gonadotroph, somatotroph, thyreotroph) or defined as plurihormonal in the case of overproduction of more than one hormone. Tumors lacking a pronounced hormone level of one type of hormones are classified as hormone-inactive tumors (silent or null cell adenomas). However, the distribution of tumor types documented in surgical, radiological, histopathological or endocrinological studies varies greatly. According to a meta-analysis of Ezzat et al., hormone-producing adenomas account for 65%, and hormone-inactive for 35% [3,4]. Generally, these tumors are benign and slow-growing adenomas. Their clinical importance lies in the following dysfunction of various organs caused by imbalanced hormone production. The standard procedures for diagnosing pituitary adenomas includes magnetic resonance imaging (MRI) followed by trans-nasal surgical resection, with the goal of the treatment being the seamless removal of tumor tissue while preserving healthy gland function. However, an unambiguous discrimination between gland tissue and adenoma is not always explicitly possible via visual inspection during surgery. Invasive growth of the tumor into circumjacent tissue often hinders precise resection of the pituitary adenoma. In the case of insufficient surgical tumor removal, recurrence will occur in pituitary adenomas. Even after gross total resection, remission is dependent on the growth pattern, size and subtype of pituitary adenomas [5]. Insufficient removal therefore causes recurrence of the tumor leading to follow-up surgeries, drug therapies or radiosurgical treatment. On the other hand, excess resection of healthy pituitary gland tissue can result in the requirement for hormonal substitution therapy to maintain correct organ function. As current approaches are limited in the detection of borders between pituitary gland and adenoma, additional techniques are sought to assist surgeons in distinguishing gland tissue from pituitary adenoma to seamlessly remove tumorous tissue while preserving gland tissue, maintaining healthy pituitary gland function.

Spontaneous Raman spectroscopy has been widely applied to the medical field [6,7,8,9]. Raman spectra yield vast information about the molecular composition of tissue, discriminating tissue types based on their molecular characteristics. Several studies have shown that, depending on molecular differences, healthy tissue exhibits a different Raman spectrum to tumorous tissue, making this technique attractive for diagnostic purposes [8,10,11,12]. Zhou et al. demonstrated resonant Raman spectroscopy on brain tumors and benign pituitary adenoma [13], however this study did not differentiate different types of pituitary adenoma.

Line scan Raman microspectroscopy (LSRM) [14,15,16] is a powerful instrument not only to collect Raman spectra but also to gain information about the molecular spatial distribution in 2D. Faster acquisition times compared to conventional point scan Raman spectroscopy is the main advantage of LSRM [17,18]. As LSRM scans the specimen with a laser line instead of a single laser spot, acquisition times can be reduced as multiple Raman spectra are detected simultaneously. Schlücker et al. [17] demonstrated the improvement of acquisition speed with LSRM compared to point scan Raman spectroscopy by an order of magnitude.

A related technique to Raman spectroscopy is Fourier transform infrared (FT-IR) spectroscopy [19] which has been applied to pituitary gland and pituitary adenoma by Banas et al. [20] and Steiner et al. [21]. These studies demonstrated the feasibility of FT-IR to provide diagnostic findings, however, detailed information for different pituitary adenoma types is yet to be shown.

In our study, we cover classification of different types of pituitary adenomas and gland tissues as well as the periosteal layer, the structure covering the pituitary gland. Our research demonstrates the feasibility of applying Raman spectroscopy in combination with principal component analysis (PCA) and a k-nearest neighbor (kNN) classification to pituitary adenoma diagnostics. Moreover, LSRM provides additional insight into the spatial distribution of molecular compounds including lipids, proteins, collagen, carbohydrates and phenylalanine. Quantification of LSRM images was performed using texture analysis, calculating both grey level cooccurrence matrices (GLCMs) of each LSRM image and correlation coefficients between LSRM images.

## 2. Results and Discussion

Our dataset comprised 28 biopsies originating from 20 patients which were investigated by our LSRM system. Histopathological analysis, provided by neuropathologists, was available for all biopsies to validate our findings. The distribution of the biopsies was divided into the following: eight biopsies of gland tissue, five gonadotroph, six null cell, three corticotroph, three plurihormonal and two somatotroph biopsies. Additionally, we examined one sample of periosteal layer to test the performance of our classifier as periosteal layer is the circumventing tissue and consists of a different molecular structure. Its Raman signal is therefore expected to be clearly different to both pituitary gland and adenoma tissue. First, spectral analysis of all Raman spectra was performed by PCA for determination of important Raman bands and dimension reduction. To categorize the Raman spectra according to their respective classes (pituitary gland, adenoma subtypes and periosteal layer), a kNN algorithm was trained based on the principal components derived from PCA explaining 95% of variance. LSRM images were investigated at Raman peaks which were determined by the first principal component describing 76% of variance. Quantification of the differences between gland tissue and each class of adenomas, as well as periosteal layer, was achieved by applying texture analysis to calculate intra-pixel related characteristics by evaluating GLCMs followed by 2D correlation coefficient analysis for comparison of LSRM images of pituitary gland, adenomas and periosteal layer samples.

### 2.1. Raman Spectral Analysis

**Figure 1:**
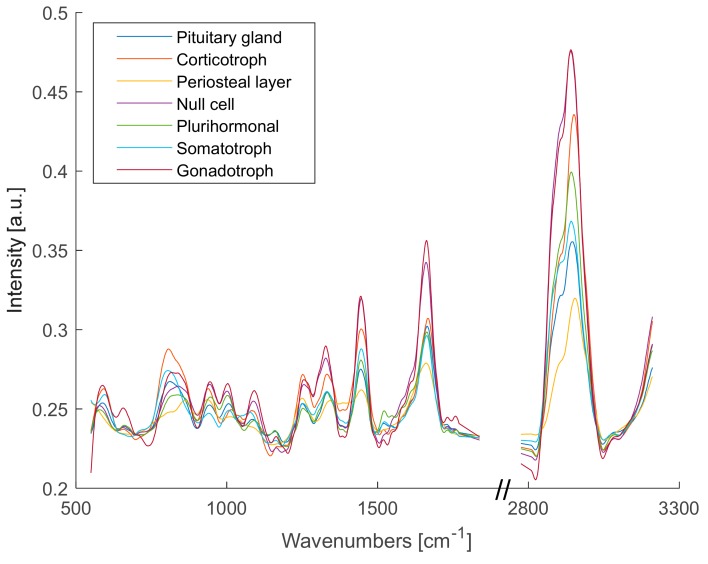
Medians of Raman spectra of pituitary gland tissue, adenoma subtypes (corticotroph, null cell, plurihormonal, somatotroph, gonadotroph) and periosteal layer.

Figure 1 shows the medians of the Raman spectra of pituitary gland tissue, adenoma subtypes and periosteal layer. To provide a detailed overview of the fingerprint region (500–1700 cm^−1^) and the CH-stretching region (2800–3000 cm^−1^), the silent region in between was left out as it did not contain any information about differences between the Raman spectra. PCA was used to decompose all Raman spectra into their loading vectors explaining the significant differences between gland tissue, adenoma subtypes and periosteal layer.

**Figure 2:**
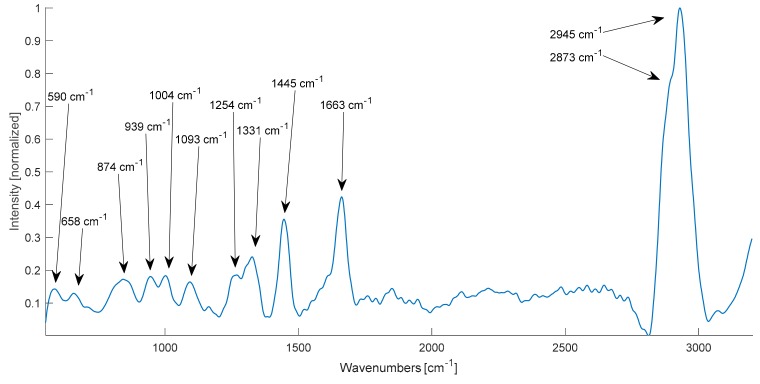
First principal component calculated via PCA with labelled Raman bands.

PCA could identify the Raman bands with the largest variances. The first principal component, accounting for 76% of variance, is shown in Figure 2 and the following peaks are highlighted: 590 cm^−1^, 658 cm^−1^, 874 cm^−1^, 939 cm^−1^, 1004 cm^−1^, 1093 cm^−1^, 1254 cm^−1^, 1331 cm^−1^, 1445 cm^−1^, 1663 cm^−1^ and in the CH_2_/CH_3_-stretching region at 2873 and 2945 cm^−1^. The labels in Figure 2 indicate the Raman peaks which were further used to visualize LSRM images. Assignments to these peaks could be correlated with previously identified Raman bands by literature and an overview can be found in Table 1 [8,13,22,23,24,25].

The Raman peak at 590 cm^−1^ could be assigned to the phospholipid phosphatidylinositol as this vibration is correlated to the stretching of PO_4_. Tyrosine and its precursor phenylalanine were attributed to the Raman bands at 658 and 1004 cm^−1^, as these bands are typical for phenylalanine in general. Prominent bands for proteins and lipids could be found at 1445 cm^−1^, 1663 cm^−1^ and in the lipid region at 2873 and 2945 cm^−1^. The combination of protein and collagen was assigned to the Raman peaks located at 939, 1254 and 1331 cm^−1^. In addition to protein and collagen, glycogen was assigned to 874 cm^−1^. The Raman band at 1093 cm^−1^ was explained by the vibrational modes ν(PO_2_^−^), ν(C-C), ν(C-N) attributed to nucleic acids, phospholipids, glycogen and collagen.

Feeding the Raman spectra into a kNN classifier upon PCA, gland tissue could be differentiated from adenomas. In total, 64,087 Raman spectra were analyzed in this study. For the kNN classification, 15 principal components were kept explaining 95% of variance. The first three principal components covered 76%, 7% and 3% of variance, respectively. To avoid overfitting, a holdout validation with 25% held out was chosen. Table 2 provides an overview of the number of investigated biopsies and the respective accuracies. The performance of the kNN algorithm yielded high accuracies in classification of gland tissue, hormone-producing and hormone-inactive adenoma types, and periosteal layer. For pituitary gland tissue, the kNN algorithm achieved 95% accuracy. In case of hormone-producing adenomas, the recorded accuracies were 84% for corticotroph adenoma, 88% for gonadotroph adenoma, and 99% for somatotroph adenoma. The comparably lower accuracies for corticotroph and gonadotroph adenoma could be explained by low molecular specificity. However, the accuracies for these two hormone-producing adenoma subtypes could be improved by either a larger dataset or increasing the number of principal components to enlarge the variance of the spectral data at cost of computational speed. Furthermore, the kNN algorithm achieved an accuracy of 91% for hormone-inactive tumors, null cell adenomas, as well as for plurihormonal adenomas. The periosteal layer could be unambiguously discriminated as the kNN yielded 100% accuracy. The explicit discrimination could be explained by the different molecular structure as this tissue is collagen richer than pituitary tissue. An overall accuracy of 92% was achieved.

Raman spectroscopy provided excellent sensitivity to molecular changes in the pituitary tissue, hence Raman spectral analysis showed pronounced differences between pituitary gland and adenoma tissue. Additional classification of different states of hormone-producing, namely corticotroph, gonadotroph, somatotroph and plurihormonal, and hormone-inactive tumors, null cell adenomas, based on molecular differences in tissue could be performed. Moreover, Raman spectra of the periosteal layer showed the robustness of the classifier. The periosteal layer exhibited a distinct Raman signature due a different molecular composition of the tissue and could be explicitly differentiated from pituitary gland and adenomas. As described in the Materials and Methods section, the Raman spectra were pre-processed following an established protocol [26].

### 2.2. Line Scan Raman Microspectroscopy Image Analysis

**Figure 3:**
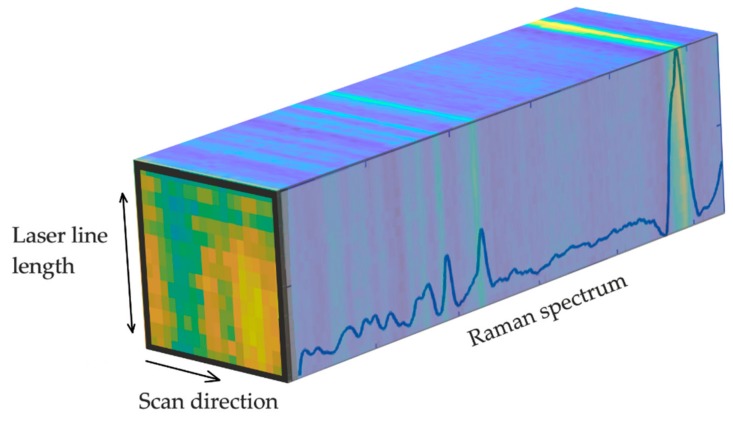
Hyperspectral cube containing Raman spectra collected across the laser line at each scan position. At Raman peaks of interest, images could be visualized providing the spatial distribution of molecular compounds. The LSRM image at the front of the hyperspectral cube shows the spatial distribution of functional molecular groups associated to the peak located at 2870 cm^−1^ of pituitary gland tissue.

As our LSRM system scanned a laser line across the pituitary samples, Raman spectra were detected along the laser line at each scan step. In Figure 3 the collected data is visualized as a hyperspectral cube, where LSRM images correspond to Raman bands of interest. LSRM images based on significant Raman bands determined by the first principal component of the PCA were visualized. Spatially resolved intensity differences given by spectral analysis visualized the distribution of molecular compounds and assisted in further investigation of pathologic characteristics.

#### 2.2.1. Line Scan Raman Microspectroscopy Images

**Figure 4:**
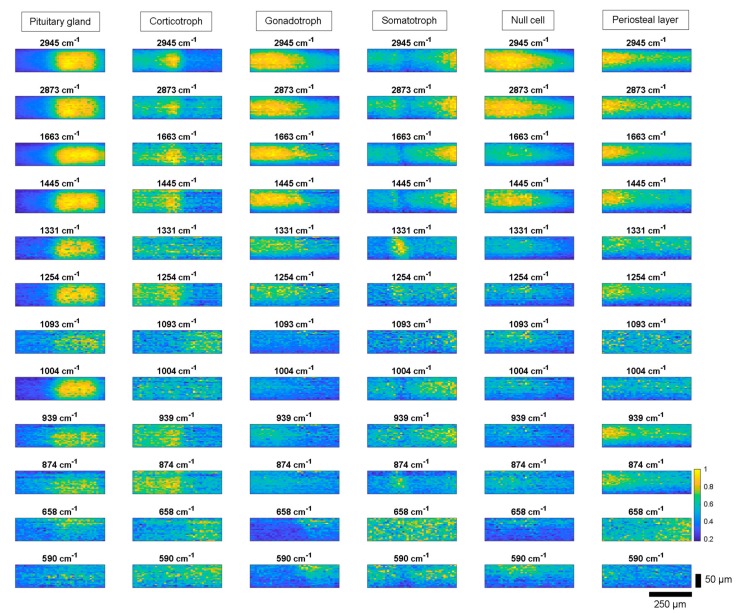
LSRM images at representative Raman peaks based on Raman spectral intensity for pituitary gland, corticotroph, gonadotroph, somatotroph and null cell adenomas as well as periosteal layer. The visualized Raman bands were determined based on the first principal component of the PCA. Differences in intensity levels and spatial distribution of functional molecular groups between pituitary gland, adenoma subtypes and periosteal layer were investigated. Scale bar: horizontal: 250 µm, vertical: 50 µm.

Figure 4 shows representative LSRM images per class (pituitary gland, adenoma tissue and periosteal layer) visualized at distinguishable Raman bands based on the Raman spectral intensity. For each class one representative dataset was chosen. The significance of these Raman peaks has been determined by PCA as being explained in the first principal component as shown in Figure 2. The spatially resolved intensity differences given at the different Raman peaks describes the local distribution of functional molecular groups, according to Table 1. 

**Figure 5:**
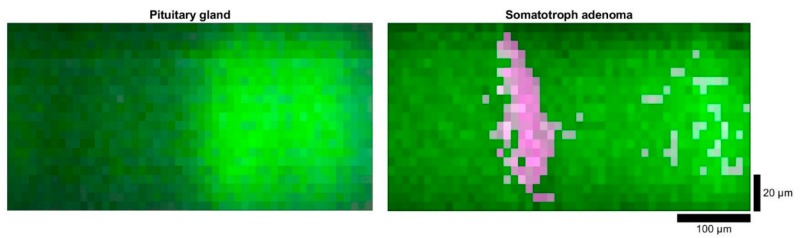
LSRM images of pituitary gland (left) and somatotroph adenoma (right): the spatial intensity differences are given by the Raman bands at 1331 cm^−1^ (in magenta) and 1445 cm^−1^ (in green). Scale bar: horizontal: 100 µm, vertical: 20 µm.

For diagnostic purposes, differences significant for each type of adenoma were determined. All adenoma types lack a Raman signal at 1004 cm^−1^, indicating a lack of phenylalanine. Gland tissue showed high Raman signal levels across all investigated Raman peaks (see first column in Figure 4). However, at lower wavenumbers, from 590 to 939 cm^−1^, and at 1093 cm^−1^ only small Raman signals were observed. Corticotroph adenoma did not exhibit pronounced spatially resolved intensity differences compared to gland tissue, see second column Figure 4. In the CH-stretching region, the Raman bands describing signals at 2873 and 2945 cm^−1^ yielded slightly different spatial intensity differences compared to gland tissue and other adenomas. Gonadotroph adenoma did not express Raman signal at 1093 cm^−1^, see third column Figure 4. At higher wavenumbers the investigated Raman bands of gonadotroph adenoma showed characteristics resembling gland tissue. Somatotroph adenoma, see fourth column, demonstrated varying contrast at different bands. At the Raman bands 1445 cm^−1^, 1663 cm^−1^, 2873 cm^−1^ and 2945 cm^−1^, high Raman counts were detected in the same tissue region, whereas Raman peaks at 874, 1254 and 1331 cm^−1^ expressed Raman counts up to 1 in different tissue areas. Figure 5 shows this effect in a color-coded image highlighting two Raman bands. Null cell adenoma demonstrated similar behavior to gland tissue in the lipid region, however the signal levels for lower wavenumbers are lower compared to pituitary gland, see fifth column Figure 4. The Raman band 1663 cm^−1^ could be used to identify null cell adenoma as it showed a different behavior compared to gland tissue and all other adenoma types. Periosteal layer expressed fewer Raman bands compared to gland tissue, exhibiting no Raman signal at Raman bands 590 cm^−1^, 658 cm^−1^, 1004 cm^−1^ or 1093 cm^−1^, see sixth column Figure 4.

Figure 5 shows LSRM images with spatially resolved differences between pituitary gland and somatotroph adenoma at the Raman bands 133^1^ and 1445 cm^−1^. There is a significant change in the spatial distribution between the two Raman bands (1331 and 1445 cm^−1^) opposing in the LSRM images of somatotroph adenoma and pituitary gland. As the signal in the LSRM image of pituitary gland originated from the same region, the spatial intensity differences did not reveal distribution variance of molecular compounds investigated at these Raman peaks. The magenta region indicates high Raman signal at 1331 cm^.1^, whereas the green region shows high levels at 1445 cm^−1^.

#### 2.2.2. Texture Analysis with Grey Level Cooccurrence Matrices

**Figure 6:**
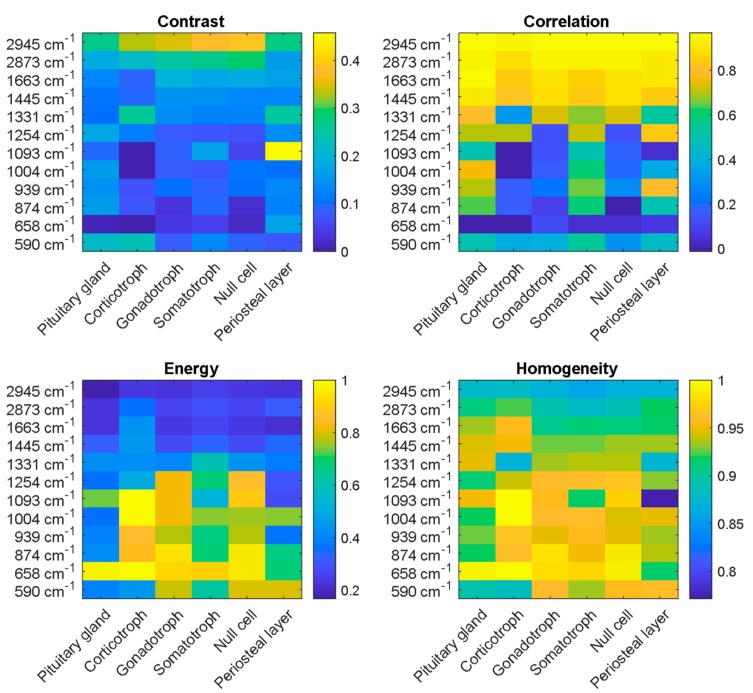
Texture analysis based on GLCM deriving the four statistical measures (contrast, correlation, energy and homogeneity) was applied to LSRM images at significant peaks determined by PCA. The four investigated properties of the GLCM describe characteristics of local variations in the GLCM (contrast), joint probability occurrence of given pixel pairs (correlation), sum of squared elements in the GLCM (energy) and distance of the distribution of entries in the GLCM to the GLCM diagonal (homogeneity). Based on the ensemble of statistical values at different wavenumbers, the investigated classes could be discriminated.

The potential to discriminate pituitary gland, adenoma subtypes and periosteal layer by LSRM images was investigated calculating the GLCM for each LSRM image at each Raman band of interest determined by PCA, for each pathological class. Thereby, statistical measures for relationships between the pixels in each image at each band were derived. As the GLCM calculates values for contrast, correlation, energy and homogeneity, it can be used to provide a first insight about the differences between the classes. Figure 6 shows the matrices visualizing the respective properties of the GLCM applied to LSRM images shown in Figure 4. In the upper left panel, the comparison of calculated values for the contrast is given for gland tissue, adenoma subtypes and periosteal layer. Contrast explaining the local variations in the GLCM exhibited in general lower values than the other statistical measures, except for the lipid band at 2945 cm^−1^. The calculated values for contrast ranged from 0 to 0.45. High contrast was observed in the lipid region at the Raman peak at 2945 cm^−1^ for corticotroph, gonadotroph, somatotroph and null cell adenoma ranging from 0.35 to 0.38. At other Raman bands, the LSRM images exhibited low contrast yielding values of 0–0.25. However, for periosteal layer we observed a high contrast at 1093 cm^−1^ ranging up to 0.45. The values calculated for the correlation, describing the joint probability occurrence of given pixel pairs, and the energy, yielding the sum of squared elements in the GLCM between pixel pairs, are given in the upper right panel of Figure 6. The correlation yielded high levels for the lipid region at 2945 and 2873 cm^−1^, ranging from 0.8 to 1. In the fingerprint region, the correlation values demonstrated a large variance, ranging from 0 to 0.8. Correlation exhibited opposing characteristics to energy in the fingerprint and CH-stretching region. The statistical value energy (providing the sum of squared elements in the GLCM is shown in the lower left panel of Figure 6) yielded low values ranging from 0.2 to 0.4 for the lipid region as well as high wavenumbers in the fingerprint region. The energy was comparably high for corticotroph, gonadotroph and null cell adenoma in the fingerprint region ranging from 590 to 1254 cm^−1^ as values up to 1 were calculated. The fourth measure derived from the GLCM covered the homogeneity of the inspected LSRM images at the given Raman bands of interest, see lower right panel of Figure 6. Homogeneity described the closeness of the distribution of entries in the GLCM to the GLCM diagonal. The homogeneity exhibited values ranging from 0.8 to 1 indicating a homogeneity among LSRM images. The homogeneity of LSRM images did not express large variances; the calculated values ranged from 0.8 to 1. Hence, the discrimination of pituitary gland and periosteal layer versus pituitary adenoma was feasible, however deeper analysis is required as adenoma subtypes expressed similar characteristics.

#### 2.2.3. Image Analysis with Correlation Coefficients

**Figure 7:**
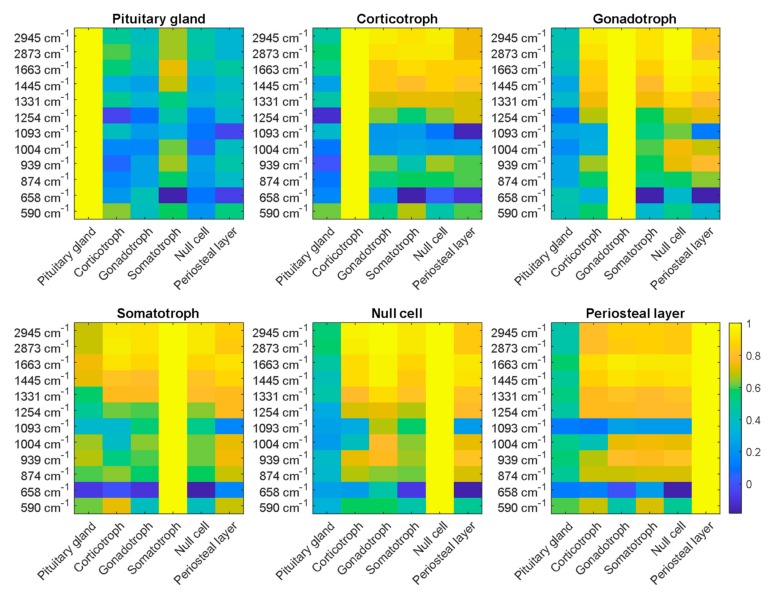
Correlation coefficients were calculated between LSRM images of pituitary gland, periosteal layer and adenoma subtypes (corticotroph, gonadotroph, somatotroph and null cell) at representative wavenumbers. The significance of these peaks was determined via the results of the PCA. Pituitary gland could be clearly discriminated based on the correlation coefficients between the different classes. As indicated on the color bar on the right, the correlation coefficient ranges from 0 to 1, where 1 quantifies high correlation between the investigated classes.

Following the results of the statistical analysis of the GLCM, correlation coefficients explaining the correlation between LSRM images at each significant Raman peak of each pathological type were calculated. This allowed an investigation into the relationship not only between pixels within one LSRM image but also between whole LSRM images of the different pathologies versus gland tissue and periosteal layer, as shown in Figure 7. Correlation coefficients between LSRM images were chosen as the method for analysis as correlation showed the most pronounced differences between pituitary gland, adenomas and periosteal layer. In general, gland tissue could be clearly classified and distinguished, whereas adenoma subtypes expressed similar characteristics. Pituitary gland tissue (plot 1 in Figure 7) was clearly discriminable from all other classes based on the correlation coefficient yielded from the LSRM analysis. Somatotroph adenoma yielded lower correlation coefficients in the lipid region compared to the other adenoma classes. However, at the wavenumbers 1663, 1445 and 1004 cm^−1^, somatotroph adenoma showed correlation coefficients of 0.6 comparable to gland tissue, whereas the other adenoma types did not express these characteristics. Corticotroph adenoma expressed similar lipid region levels compared to other types of adenoma. The correlation coefficients ranged from 0.82 to 0.93 for corticotroph adenoma in the CH-stretching region. At lower wavenumbers (874–1254 cm^−1^), the correlation coefficient decreased to 0.6 indicating higher variability between the classes. Based on the correlation coefficient, corticotroph adenoma could be clearly differentiated from gland tissue. The correlation of gonadotroph adenoma and null cell adenoma yielded a high similarity in the wavenumbers 2945, 2873, 1663 and 1445 cm^−1^ as the correlation coefficient equaled 1. The discrimination from gland tissue was unambiguous as the correlation coefficients were smaller than 0.4. Null cell adenoma did not express any correlation with gland tissue, hence LSRM images could be discriminated based on the correlation coefficient. The highest correlation with gland tissue was found in somatotroph adenoma, with correlation coefficients up to 0.8. The periosteal layer yielded high correlation with the adenoma subtypes as the correlation coefficient ranged from 0.78 to 0.91. However, at two Raman bands, 1093 cm^−1^ and 658 cm^−1^, the correlation coefficient of periosteal layer exhibited low levels ranging from 0 to 0.2.

Based on the explicit ensembles of correlation coefficients for each class, LSRM images could be classified into pituitary gland versus pituitary adenoma but not adenoma subtypes. Nonetheless, LSRM images could visualize the spatial distribution of molecular compounds of regions of interest. Hence, differences not only in spectral data could be demonstrated but also in spatial distribution characterized by textural features.

As the kNN explicitly discriminated periosteal layer from the other investigated biopsies, a more pronounced difference in correlation coefficients was expected. However, we were investigating differences explained only by the first principal component determined by PCA as this loading vector covered 76% of variance. The first principal component allowed for clear discrimination of pituitary gland and pituitary adenomas. More detailed differences between the different types of adenoma could be achieved by examining more principal components. Nonetheless, classification into the correct adenoma subtypes could be achieved by the ensemble of correlation coefficients at different wavenumbers.

Future investigations could target more detailed molecular pathology based on LSRM images providing high resolution images. Furthermore, LSRM images could assist histopathology to speed up the process of diagnostic findings leading to earlier treatment and a reduction in the number of recurring surgical interventions. An endoscopic approach could be applied for in vivo diagnosis in real time during surgery leading to higher preservation of normal pituitary gland function and, at the same time, a reduction in the number of postoperative interventions such as re-operations, drug therapy or radiosurgery.

## 3. Materials and Methods

### 3.1. Pituitary Tissue and Ethics

Pituitary specimens including adenomas and gland tissue, as well as periosteal layer samples, were provided by the Department of Neurosurgery of the Medical University of Vienna (MUV). This study including the procedures were approved by the ethics committee of the MUV (code: EK1286/2018). Prior to enrollment into the trial, all participants gave their written informed consent.

The measurements of the pituitary gland including adenoma tissue were performed at room temperature within one hour after resection or on thawed cryogenic frozen samples (−80 °C) which were frozen immediately after surgery and thawed to room temperature before measurement. The sizes of the biopsies ranged from 1 to 2 mm in diameter for pituitary gland samples to 2 mm to 5 mm for adenoma tissue. The periosteal layer sample was approximately 5 mm in diameter. After Raman investigation, the tissues were transferred to the Institute of Neurology immediately for further histopathological analysis by neuropathologists. The histopathological finding was available and provided for each biopsy.

To minimize background signals from microscope slides and interference with the Raman signals originating from gland samples, quartz slides (Alfa Aesar, Thermofisher, Waltham, MA, USA) were used for mounting the biopsies for imaging. Data were collected from different locations within the biopsies to investigate the entire sample and to account for inhomogeneities. Determination of the diagnosis of the pituitary gland was achieved by molecular analysis of Raman spectra using PCA and a classification via a kNN.

### 3.2. Line Scan Raman Microspectroscopy System

**Figure 8:**
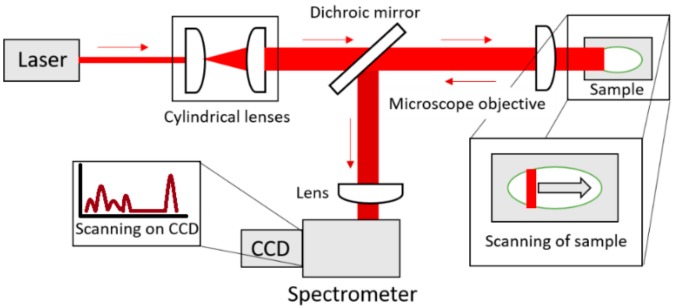
Illustration of LSRM (top view). A laser line is formed by two cylindrical lenses and focused via a microscope objective onto the sample. A translational stage moves the sample. The microscope objective collects the Raman signal from the sample in the backward direction. The Raman signal is separated from the laser light with the dichroic mirror. Focusing into the spectrometer is achieved with an additional lens.

A 785 nm diode laser (VBG-Stabilized Single Laser Source, LS−1-78-1-FA), a microscope objective (20×, NA 0.5, Zeiss, Oberkochen, Germany) and a spectrometer (Shamrock SR 303i, Andor Technology, Belfast, Northern Ireland) equipped with a CCD-camera with 255 × 1024 pixels (Newton 920i, Andor Technology, Belfast, Northern Ireland) formed the core of the LSRM system illustrated in Figure 8. The maximum output power of the laser was 600 mW while the power on the sample was held at 3 mW/µm^2^. A laser line was formed using 2 cylindrical lenses (70 mm and 40 mm, Thorlabs, Newton, NJ, USA). Filters to remove side-lobes and the excitation wavelength from the Raman signal comprised a laser clean-up filter (785 nm MaxLine^®^, LL01-785-12.5, Semrock Rochester, NY, USA) and ultra-steep long-pass edge filter (785 nm RazorEdge^®^, LP02-785RE-25, Semrock Rochester, NY, USA), respectively. A long-pass dichroic mirror with a cut-on wavelength of 805 nm (DMLP805, Thorlabs, Newton, NJ, USA) achieved the separation of Raman signal and excitation laser. A 300 l/mm grating blazed at 500 nm implemented in the spectrometer provided a spectral resolution of 0.5 nm. The slit size of the spectrometer was set to 100 µm. The integration time for each line was 10 s and the delay of the stage was 1 s, hence 5 line scans were acquired per minute.

By moving the biopsies with a linear stage (X-LSM050A-E, Zaber, Vancouver, Canada) with a step size of 10 µm and scanning the laser line with a resolution of 5 µm across the sample, Raman spectra were simultaneously collected along the laser line covering an area of 100 µm × 500 µm in 10 min (100 µm corresponding to the length of the laser line in x-direction and a perpendicular stage movement of 500 µm.)

Wavelength-calibration of the LSRM was achieved with Dimethylsulfoxid (DMSO) and paracetamol [22,27,28].

### 3.3. Raman Spectral Analysis

The Raman spectra vectors covered a range of 500–3200 cm^−1^. Raw Raman spectra were first corrected by the system transmission function taking the wavelength dependent losses of the optical components and quantum efficiency of the detector into account. Following the established protocol by Bocklitz et al. [26], Raman spectra were corrected for autofluorescence via an iterative polynome fit (polynome degree: 9) and subtraction, applying an algorithm developed in [29]. Smoothing of the Raman spectra was achieved via Savitzky–Golay filtering [30] using a smoothing window of 21 spectral sampling points and a polynome of the 3rd order. A 0–1 normalization was chosen to pre-process the data to be fed into a PCA algorithm implemented in MATLAB (MATLAB2019a, MathWorks). PCA is an orthogonal transformation determining new orthogonal basis vectors to reduce a high dimensional object into lower dimensions [31,32]. Each new basis vector covers a variance of the dataset in descending manner. The first new basis vector describes the largest variance followed by the second new basis vector explaining smaller variances. The fine differences are described by the following new basis vectors. These basis vectors are also known as principal components.

For the kNN classification, we used the application *Classification Learner* included in the toolbox *Statistics and Machine Learning 11.5* by MATLAB. The kNN algorithm classifies based on the number of given nearest datapoints and the distance between datapoints [33,34,35]. The fine kNN showed the discrimination of the different pathological classes compared to gland tissue and periosteal layer. Properties of the kNN included Euclidian distances and equal distance weights. The classifier was set to one neighbor.

### 3.4. LSRM Image Visualization and Analysis

As the PCA on Raman spectra provided information on the bands of the largest variance, images of the pituitary tissue were visualized at these bands of interest. The first principal component described 76% of variance and images were visualized at indicated peaks in the hyperspectral cube.

Texture analysis was implemented in MATLAB, following the algorithm calculating GLCMs developed by Haralick et al. [36]. The GLCM functions *graycomatrix* and *graycoprops* were used to calculate statistical features from Raman maps at the given Raman peaks which were determined by PCA. As the GLCM calculates the statistics for adjacent pixels, four different offsets were used to ensure the statistical relevance within each analyzed LSRM image, namely 0, 45, 90 and 135 degrees. The statistical measures which can be derived from the GLCM include contrast, correlation, energy and homogeneity. The contrast parameter provides the intensity difference between a pixel and its neighbor. In case of a constant image, the contrast yields 0 as a constant image only exhibits one value over the entire array. Correlation indicates how likely it is that two adjacent pixels are related. If an image is perfectly correlated, the correlation yields −1 for a negatively correlated image or 1 for a positively correlated image, hence values calculated for correlation range from −1 to 1. The property energy provides the statistical values covering the sum of squared elements in the GLCM. In case of a constant image the energy equals 1. Valid values for energy range from 0 to 1. Other names for energy include uniformity, uniformity of energy and angular second moment. The statistical measure homogeneity calculates the distance of the distribution of entries in the GLCM to the GLCM diagonal. If the GLCM is a diagonal matrix, the homogeneity equals 1. The range for values for homogeneity ranges from 0 to 1. These values were calculated for each LSRM image of each pathological class at a Raman peak of interest followed by determining the median as shown in Figure 6. As we investigated 12 Raman peaks of interest determined by the first principal component of the PCA for each of the six pathological classes, we obtained 72 statistical values.

Correlation coefficients were calculated using the *corr2* function in MATLAB which provides the 2D correlation coefficient of two given arrays. The correlation coefficient describes how likely two arrays are to be the related. Two identical images yield a correlation coefficient of 1. Using correlation coefficients for hyperspectral image analysis has been widely applied [37].

## 4. Conclusions

In this study, we demonstrated the classification of pituitary gland, different types of pituitary adenomas (including hormone-producing and hormone-inactive tumors) and a periosteal layer sample based on molecular differences identified by Raman spectra with PCA followed by a kNN algorithm. The results show the feasibility of Raman spectroscopy to provide diagnosis with high accuracy. The achieved accuracies of 95% for pituitary gland, 100% for periosteal layer and 84% to 99% for adenoma subtypes indicate the potential of Raman spectroscopy for much faster pituitary adenoma diagnostics when compared to conventional histopathology. Additionally, LSRM could visualize molecular-resolved images of specific regions of interest to unravel information about the spatial distribution of different molecules in the specimen. Based on texture analysis within LSRM images calculating contrast, correlation, energy and homogeneity, properties of each LSRM image were statistically quantified providing thumbprints specifying each investigated class. To identify characteristics between LSRM images of analyzed classes, correlation coefficients were derived. Properties found by these approaches facilitated further discrimination of the classes. Raman signals could therefore be measured in situ providing instant diagnosis on the state of the inspected tissue. Future experiments in endoscopic settings could potentially pave the way towards in vivo diagnosis.

## Figures and Tables

**Table 1:** Raman band assignments of vibrational modes at significant Raman peaks determined by the first principal component derived from PCA.

Raman Shift [cm^−1^]	Vibrational Mode *	Assignment
590	ν(PO_4_)	Phosphatidylinositol
658	τ(C-C)	Phenylalanine, tyrosine, proteins
874	δ_d_(CCH), ν(C-C)	Carbohydrates, glycogen, protein and collagen
939	ν(C-C)	Protein, collagen backbone
1004	ν(C-C) aromatic ring breathing	Phenylalanine
1093	ν(PO_2_^-^), ν(C-C), ν(C-N)	Nucleic acids, phospholipids, glycogen, collagen
1254	Amide III (mix of ν(C-N) and δ(N-H))	α-helix, protein, collagen
1331	τ(CH_3_/CH_2_), ω(CH_3_/CH_2_)	Tryptophan, protein, collagen
1445	δ(CH_2_/CH_3_)	Fatty acids, protein, lipids
1663	ν(C=O), ν(C=C), Amide I	Unsaturated fatty acids, α-helix, protein, lipids
2873	ν(CH_2_)	Lipids
2945	ν(CH_3_)	Protein, lipids

* References: [8,13,22,23,24,25]; ν: stretching, δ: bending, ω: wagging, τ: twisting.

**Table 2:** Overview of investigated adenoma types and statistics fed into the PCA and kNN.

Pituitary Gland Tissue	Number of Biopsies	Accuracy of kNN Classification
Corticotroph	3	84%
Gonadotroph	5	88%
Somatotroph	2	99%
Plurihormonal	3	91%
Null cell	6	91%
Pituitary gland	8	95%
Periosteal layer	1	100%
